# Size dependent effects of Gold Nanoparticles in ISO-induced Hyperthyroid Rats

**DOI:** 10.1038/s41598-018-27934-9

**Published:** 2018-07-19

**Authors:** Jingwen Zhang, Yanbo Xue, Yajuan Ni, Feifei Ning, Lijun Shang, Aiqun Ma

**Affiliations:** 1 Department of Cardiovascular Medicine, First Affiliated Hospital of Xi’an Jiaotong University, Shaanxi Key Laboratory of Molecular Cardiology, Key Laboratory of Environment and Genes Related to Diseases, Ministry of Education, Xi’an, Shaanxi China; 2 Department of Cardiovascular Medicine, Second Affiliated Hospital of Xi’an Jiaotong University, Xi’an, Shaanxi China; 3 School of Chemistry and Biosciences, University of Bradford, Bradford, BD7 1DP UK

## Abstract

In this study, we applied different sizes of gold nanoparticles (Au-NPs) to isoproterenol (ISO)-induced hyperthyroid heart disease rats (HHD rats). Single dose of 5, 40, 100 nm Au-NPs were injected intravenously. Cardiac safety tests were evaluated by cardiac marker enzymes in serum and cardiac accumulation of Au-NPs were measured by ICP-MS. Our results showed that size-dependent cardiac effects of Au-NPs in ISO-induced hyperthyroid rats. 5 nm Au-NPs had some cardiac protective effect  but little accumulation in heart, probably due to smaller size Au-NPs can adapt to whole body easily *in vivo*. Histological analysis and TUNEL staining showed that Au-NPs can induce pathological alterations including cardiac fibrosis, apoptosis in control groups, however they can protect HHD groups from these harmful effects. Furthermore, transmission electron microscopy and western blotting employed on H9C2 cells showed that autophagy presented in Au-NPs treated cells and that Au-NPs can decrease LC3 II turning to LC3 I and decrease APG7 and caspase 12 in the process in HHD groups, while opposite effects on control groups were presented, which could be an adaptive inflammation reacts. As there are few animal studies about using nanoparticles in the treatment of heart disease, our *in vivo* and *in vitro* studies would provide valuable information before they can be considered for clinical use in general.

## Introduction

Hyperthyroid heart disease is characterized as high cardiac output and dynamic ECG changes. It is usually caused by high sensitivity of the heart to catechomale. Long-term untreated hyperthyroid heart disease can lead to heart failure, which is hard to treat and have poor prognosis. Currently there is no effective methods to treat end-stage heart failure, therefore finding a new way to target the heart in the first place is still a best prevention.

Gold Nanoparticles (Au-NPs) have been widely employed in biomedical fields, such as imaging and biological labeling^1^, cancer treatment^2,3^, and their toxic effects on the liver, lung, kidney, brain and reproductive system have been studied widely^4–8^. There are also growing interests to investigate its effects on the heart, but all present studies vary from the type of Au-NPs used, the different heart disease conditions and animal models employed, and the administration periods and routes *etc.*^9–14^. In general, the accumulation of Au-NPs in the heart is size-dependent when administered intravenously^15^ but the conclusion varied from case to case. Therefore, there are still great needs to have detailed studies on the effects of Au-NPs on the heart and its related diseases.

In this study, we used different sizes of 5, 40, 100 nm Au-NPs to test their effects on both normal and ISO-induced hyperthyroid rats by measuring ECG and UCG parameters and cardiac enzyme in serum. The accumulation distributions of Au-NPs in the heart was also measured by inductively coupled plasma mass spectrometry (ICP-MS). Furthermore, Histological analysis and TUNEL staining were used to determine pathological alterations including cardiac fibrosis and apoptosis. It is known that autophagy can trigger apoptosis and lead to cell death, but it can also present cardiac protective mechanism under several basal conditions. Therefore, transmission electron microscopy and western blotting were both employed on H9C2 cells (a sub-clone derived from embryonic rat heart tissue) *in vitro* to evaluate the effects of autophagy and to explore the possible mechanism of the effects of Au-NPs. We found that Au-NPs can induce protective effects on HHD groups by decreasing their autophagy levels, but increase autophagy in control groups, which could be an adaptive inflammation reacts. However, they can induce cardiac toxicity and change the cardiac function if their uses are not properly controlled. These effects of Au-NPs on heart can bring altered histological structure in the first place before they can change the cardiac function. As there are few animal studies of using nanoparticles in the treatment of heart disease, our *in vivo and in vitro* studies would provide valuable information before they can be considered for clinical use in general.

## Results

### A week Isoproterenol injection produce a hyperthyroid heart disease rat model

Hyperthyroid disease is characterized by typical symptoms of cardiac arrhythmia, including nodal tachycardia, premature atrial contraction, paroxysmal tachycardia, ventricular flutter and ventricular fibrillation. Ventricular fibrillation is the most common change among them. Hyperthyroid disease inducing to heart failure is associated with several reasons, including (1) Hyperthyroid can induce beta receptor hyperfunction in the heart. Longer time of heavy heart work load can then lead to heart enlargement and cardiac output increase; (2) Heart oxygen consumption increase and cause energy dysmetabolism; (3) Tachyarrhythmia, typically ventricular fibrillation, can induce cardiac output decrease.

Typical ECG changes of hyperthyroid heart disease include but not exclude: (1) left ventricular hypertrophy; (2) ST-T segment changes: pathological decline of ST-T segment and T wave changes (decline, bidirectional and inversion); (3) Hyperthyroidism P wave: it is known that 26% of hyperthyroid disease patients has abnormal P wave changes; (4) P-Q segment changes: 1.7~4.6% of hyperthyroid patients has P-Q segment increase; (5) High T wave: 14% of severe hyperthyroid patients has high T wave; and 6) Q-T segment: Q-T segment increase is more common than decrease.

**Figure 1:**
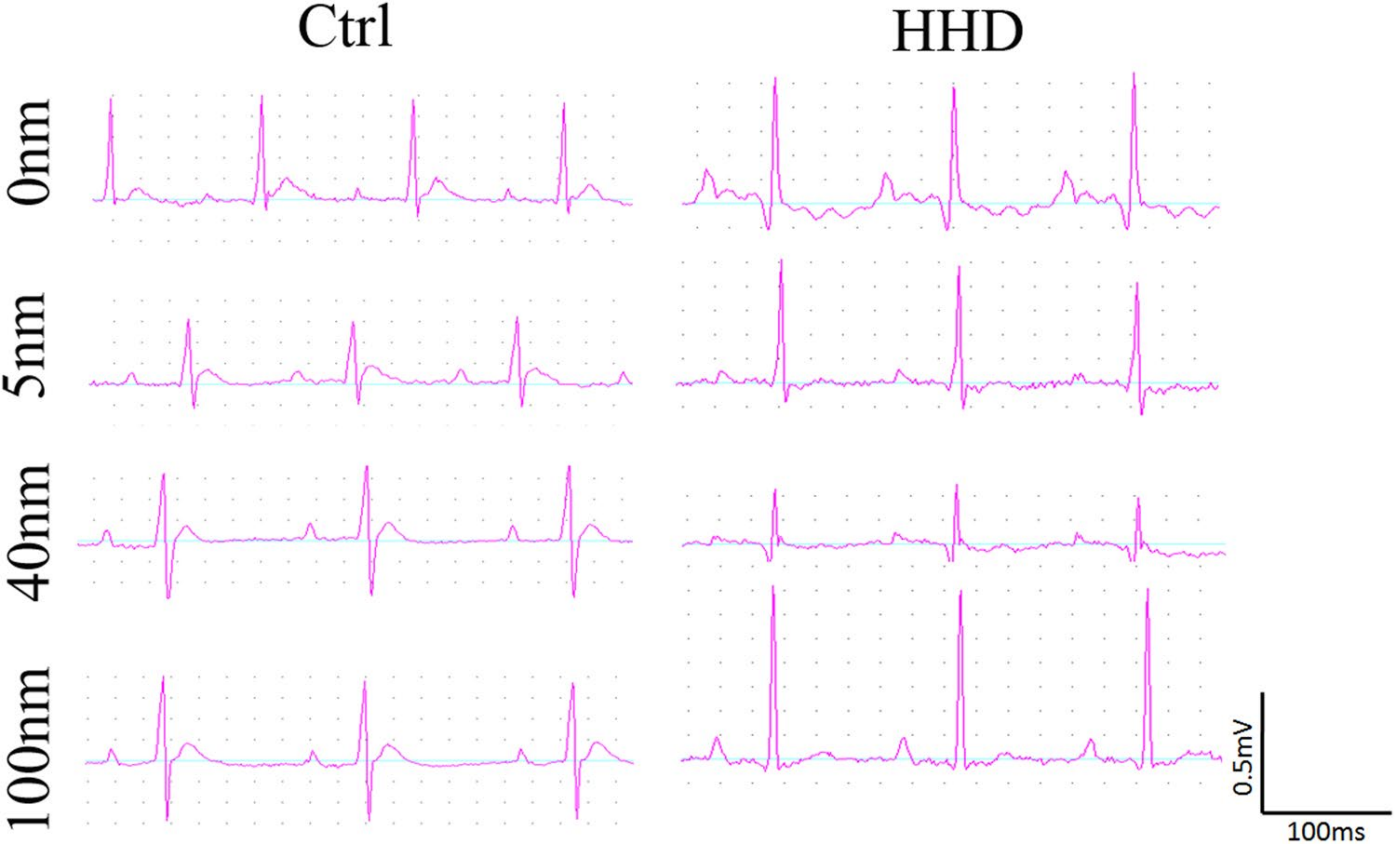
ISO-induced electrocardiogram changes mimicked hyperthyroid heart disease: changed P wave morphology and increased Q wave. The size of Au-NPs had no effect ECG morphology in normal rat hearts. Au-NPs protected against ISO-induced P wave or Q wave changes but may induce the increase in R wave amplitude.

We used a large dose of ISO (20 mg/kg/mL) intraperitoneal injection in SD rats for 7 days, and this can lead to the classic changes of hyperthyroid heart disease in electrocardiogram and measurements (Fig. 1, Table 1). There was no observation of significant changes on animal behaviors and body weights (Supplementary Fig. 1). These were evidenced by measured parameters compared to the normal control, such as raised q wave amplitude, high peak of P wave morphology (indicated by P wave amplitude/P wave duration), increased P wave amplitude, decreased P wave duration, and increased QTc interval (Table 1). All these changes indicated that ISO can induce the classic electrocardiogram phenomenon of hyperthyroid heart disease, which has high risk of cardiac arrhythmia.

**Table 1:** Electrocardiogram parameters measured in both control and hyperthyroid heart disease rats treated with different sizes of Au-NPs.

	Group	0 nm	5 nm	40 nm	100 nm
P-R segment duration (ms)	Ctrl	44.10 ± 1.79	44.67 ± 0.95	49.67 ± 1.41	44.00 ± 2.70
	HHD	39.00 ± 3.35	36.33 ± 2.32	40.29 ± 1.67	41.60 ± 1.17
QTc (ms)	Ctrl	130.12 ± 4.47	120.30 ± 11.77	113.11 ± 7.07*	129.22 ± 11.86
	HHD	151.02 ± 11.28^§^	142.06 ± 12.15	140.27 ± 5.96	149.82 ± 8.51
P wave duration (ms)	Ctrl	16.20 ± 0.63	19.67 ± 2.22*	16.83 ± 0.98	15.60 ± 2.20
	HHD	12.00 ± 0.55^§§^	12.17 ± 0.83	13.29 ± 0.75	12.60 ± 1.29
P wave amplitude(mV)	Ctrl	0.057 ± 0.00396	0.0567 ± 0.0042	0.07 ± 0.00966	0.058 ± 0.01068
	HHD	0.1120 ± 0.01356^§§§^	0.0667 ± 0.00989*	0.0643 ± 0.01251*	0.08 ± 0.00837
P (mV)/P(ms)	Ctrl	0.0036 ± 0.00030	0.0032 ± 0.00052	0.0043 ± 0.00066	0.0041 ± 0.00099
	HHD	0.0096 ± 0.00145^§§§^	0.0056 ± 0.00085*	0.0051 ± 0.00123*	0.0065 ± 0.00074
Q wave amplitude(mV)	Ctrl	0.0210 ± 0.00504	0.0217 ± 0.00543	0.0167 ± 0.00422	0.018 ± 0.002
	HHD	0.054 ± 0.02227^§^	0.0333 ± 0.00558	0.0240 ± 0.00245	0.05 ± 0.01304
QRS duration(ms)	Ctrl	25.70 ± 3.89	22.17 ± 1.33	21.17 ± 0.60	21.60 ± 0.60
	HHD	24.40 ± 2.89	22.33 ± 1.65	19.14 ± 1.01*	20.60 ± 1.57
R wave amplitude(mV)	Ctrl	0.567 ± 0.04387	0.3433 ± 0.07877*	0.4483 ± 0.07414	0.42 ± 0.02
	HHD	0.42 ± 0.12946	0.5067 ± 0.05548	0.4671 ± 0.078	0.65 ± 0.08
S wave amplitude(mV)	Ctrl	0.138 ± 0.02235	0.2533 ± 0.08019	0.1517 ± 0.04826	0.274 ± 0.05446*
	HHD	0.286 ± 0.07305^§^	0.1217 ± 0.03928*	0.1186 ± 0.04554*	0.04 ± 0.03808*
R(mV)/S(mV)	Ctrl	4.91 ± 0.68	2.46 ± 0.79	8.66 ± 4.45	2.60 ± 1.16
	HHD	2.11 ± 0.86	7.45 ± 2.71	17.16 ± 10.74	19.76 ± 10.39
T wave amplitude(mV)	Ctrl	0.075 ± 0.02001	0.0533 ± 0.02472	0.0617 ± 0.01327	0.082 ± 0.02417
	HHD	0.03 ± 0.03	0.0233 ± 0.01783	0.0157 ± 0.0173	0.044 ± 0.004
T wave duration(ms)	Ctrl	23.67 ± 2.96	28.67 ± 7.88	30.33 ± 1.86	28.67 ± 11.92
	HHD	36.33 ± 3.18^§^	18.67 ± 2.19**	31.33 ± 10.48	31.67 ± 3.76
T (mV)/T(ms)	Ctrl	0.0021 ± 0.00069	0.0015 ± 0.00121	0.0021 ± 0.00037	0.0017 ± 0.00093
	HHD	0.0012 ± 0.00138	0.0011 ± 0.00104	0.0004 ± 0.00052	0.0014 ± 0.00015

Intra-group significance, *P < 0.05, **P < 0.01, ***P < 0.001; Inter-group group significance, ^§^P < 0.05, ^§§^P < 0.01, ^§§§^P < 0.001; n = 6.

The further results of Echocardiography showed that hyperthyroid heart disease (HHD) groups had significant increase of cardiac output (CO) and atrial enlargement (left atrial longitudinal diameter), which were in accordance with the changes of hyperthyroid, heart disease on heart function and structure parameters. ECG analysis showed that HHD groups had ventricular high voltage (indicating left ventricular hypertrophy), biodirectional P wave or ventricular flutter, QT segment increase (Table 2). All the above ECG changes were in accordance with hyperthyroid heart disease. These results indicated that a large dose of ISO can successfully mimick the pathophysiological state of hyperthyroid heart disease. At the same time, these results indirectly showed that the high sensitivity of catecholamine on the heart was the primary reason of hyperthyroid heart disease at least in the early stage, while the toxicity of thyroxine on the heart ranked the second reason.

**Table 2:** Effects of Au-NPs on cardiac function and heart morphology measured by Echocardiography in normal and ISO-induced hyperthyroid heart disease groups.

	Group	0 nm	5 nm	40 nm	100 nm
EF%	Ctrl	82.62 ± 1.33	80.15 ± 2.87	76.27 ± 2.17*	82.16 ± 3.63
	HHD	85.45 ± 0.76	83.33 ± 2.8	79.58 ± 1.90*	82.40 ± 3.23
FS%	Ctrl	46.21 ± 1.36	43.92 ± 2.92	39.62 ± 2.02*	46.44 ± 3.82
	HHD	49.18 ± 0.87^§^	47.4 ± 2.8	43.2 ± 1.95*	46.6 ± 3.82
CO (L/min)	Ctrl	0.17 ± 0.01	0.11 ± 0.01**	0.10 ± 0.01***	0.13 ± 0.01*
	HHD	0.19 ± 0.01^§^	0.18 ± 0.02**	0.19 ± 0.02	0.19 ± 0.02
LVEDd (mm)	Ctrl	5.89 ± 0.13	5.36 ± 0.26	5.57 ± 0.2	5.77 ± 0.17
	HHD	6.08 ± 0.25	5.98 ± 0.17	6.16 ± 0.24	5.95 ± 0.25
LVESd (mm)	Ctrl	3.18 ± 0.14	3.05 ± 0.28	3.36 ± 0.14	3.11 ± 0.31
	HHD	3.10 ± 0.16	3.14 ± 0.2	3.51 ± 0.22	3.19 ± 0.29
EDV (mL)	Ctrl	0.48 ± 0.03	0.39 ± 0.04	0.42 ± 0.04	0.46 ± 0.04
	HHD	0.54 ± 0.06	0.5 ± 0.04	0.55 ± 0.06	0.51 ± 0.07
ESV (mL)	Ctrl	0.09 ± 0.01	0.08 ± 0.02	0.1 ± 0.01	0.09 ± 0.02
	HHD	0.08 ± 0.01	0.08 ± 0.02	0.12 ± 0.02	0.09 ± 0.02
IVSd (mm)	Ctrl	1.31 ± 0.05	1.43 ± 0.08	1.38 ± 0.05	1.19 ± 0.27
	HHD	1.22 ± 0.13	1.24 ± 0.04*	1.29 ± 0.05	1.25 ± 0.08
IVSs (mm)	Ctrl	1.97 ± 0.09	1.79 ± 0.1	1.95 ± 0.12	1.97 ± 0.13
	HHD	1.81 ± 0.12	1.80 ± 0.12	1.82 ± 0.08	1.74 ± 0.12
LVPWd (mm)	Ctrl	1.40 ± 0.03	1.40 ± 0.06	1.37 ± 0.04	1.46 ± 0.06
	HHD	1.30 ± 0.11	1.36 ± 0.05	1.35 ± 0.07	1.30 ± 0.09
LVPWs (mm)	Ctrl	2.09 ± 0.08	1.96 ± 0.11	2.06 ± 0.13	2.29 ± 0.09*
	HHD	2.01 ± 0.11	2.00 ± 0.12	1.96 ± 0.11	1.98 ± 0.13
Left atrial cross-sectional (mm)	Ctrl	4.1 ± 0.14	3.58 ± 0.16*	4.16 ± 0.16	4.28 ± 0.13
	HHD	4.06 ± 0.22	3.85 ± 0.15	4.57 ± 0.07*	4.37 ± 0.34
Left atrial longitudinal (mm)	Ctrl	4.52 ± 0.15	4.40 ± 0.18	4.90 ± 0.18	4.17 ± 0.1*
	HHD	4.99 ± 0.28	4.72 ± 0.28	5.17 ± 0.30	5.03 ± 0.35*
BP (btm)	Ctrl	406.24 ± 9.59	381.11 ± 19.73	340.51 ± 22.64*	345.48 ± 31.76*
	HHD	432.50 ± 9.62^§^	452.25 ± 10.29	441.24 ± 6.26	440.86 ± 10.36

Intra-group significance, *P < 0.05, **P < 0.01, ***P < 0.001; Inter-group group significance, ^§^P < 0.05, ^§§^P < 0.01, ^§§§^P < 0.001; n = 6.

### Au-NPs can reduce ISO induced cardiac toxicity

We further tested the cardiac effects of different sizes of Au-NPs on both control and ISO-induced hyperthyroid heart disease rats. After sedation, three leads were connected to rats’ three limbs separately. All electrocardiogram parameters were measured using BL420E recording system and listed in Table 1.

We recorded P-R segment duration and corrected QT interval in normal and HHD groups. These parameters can reflect heart conduction function and are correspondence to sinoatrial node conduction and ventricular repolarization function separately. We applied Au-NPs to ISO-induced rats and the results showed that they can affect P-R segment duration and corrected QT interval evaluation (Table 1). Au-NPs had minor effect on heart conduction function, except for 40 nm Au-NPs, which decreased QTc in normal group (Table 1), indicating 40 nm Au-NPs may interfere with ventricular repolarization process in normal rats.

The effects of Au-NPs on atrial depolarization were measured by P wave duration, P wave amplitude and their ratio. 5 and 40 nm Au-NPs can effectively improve P wave morphology by decreasing P wave amplitude (Table 1) in HHD groups, indicating 5 and 40 nm Au-NPs may interfere with atrial depolarization in hyperthyroid heart disease rats.

The effects of Au-NPs on ventricular depolarization were measured by q wave amplitude and QRS duration. Au-NPs had no effect on q wave in both control and hyperthyroid heart disease groups. QRS duration was also unchanged in HHD groups, except 40 nm-AuNPs can decrease QRS duration in HHD groups (Table 1), indicating 40 nm Au-NPs interfere with ventricular depolarization in HHD groups.

The effects of Au-NPs on ventricular depolarization were measured by R wave and S wave amplitude and their ratio. Au-NPs had minor effect on R and S wave amplitude in control groups, except 5 nm Au-NPs, which decreased R wave amplitude in normal groups (Table 1). All sizes of Au-NPs decreased ISO-induced S wave increasing in HHD groups (Table 1). Since 100 nm Au-NPs tended to increase R wave (Ctrl-100 nm: 0.42 ± 0.02 vs HHD-100 nm: 0.65 ± 0.08 mV, P < 0.05, n = 5), together with decreased S wave, 100 nm-AuNPs treated HHD rats presented left ventricular high voltage amplitude. These results indicated that 100 nm Au-NPs is more likely to induce ventricular hypertrophy, while 5 and 40 nm Au-NPs are less likely compared with 100 nm Au-NPs, at least in HHD groups.

**Figure 2:**
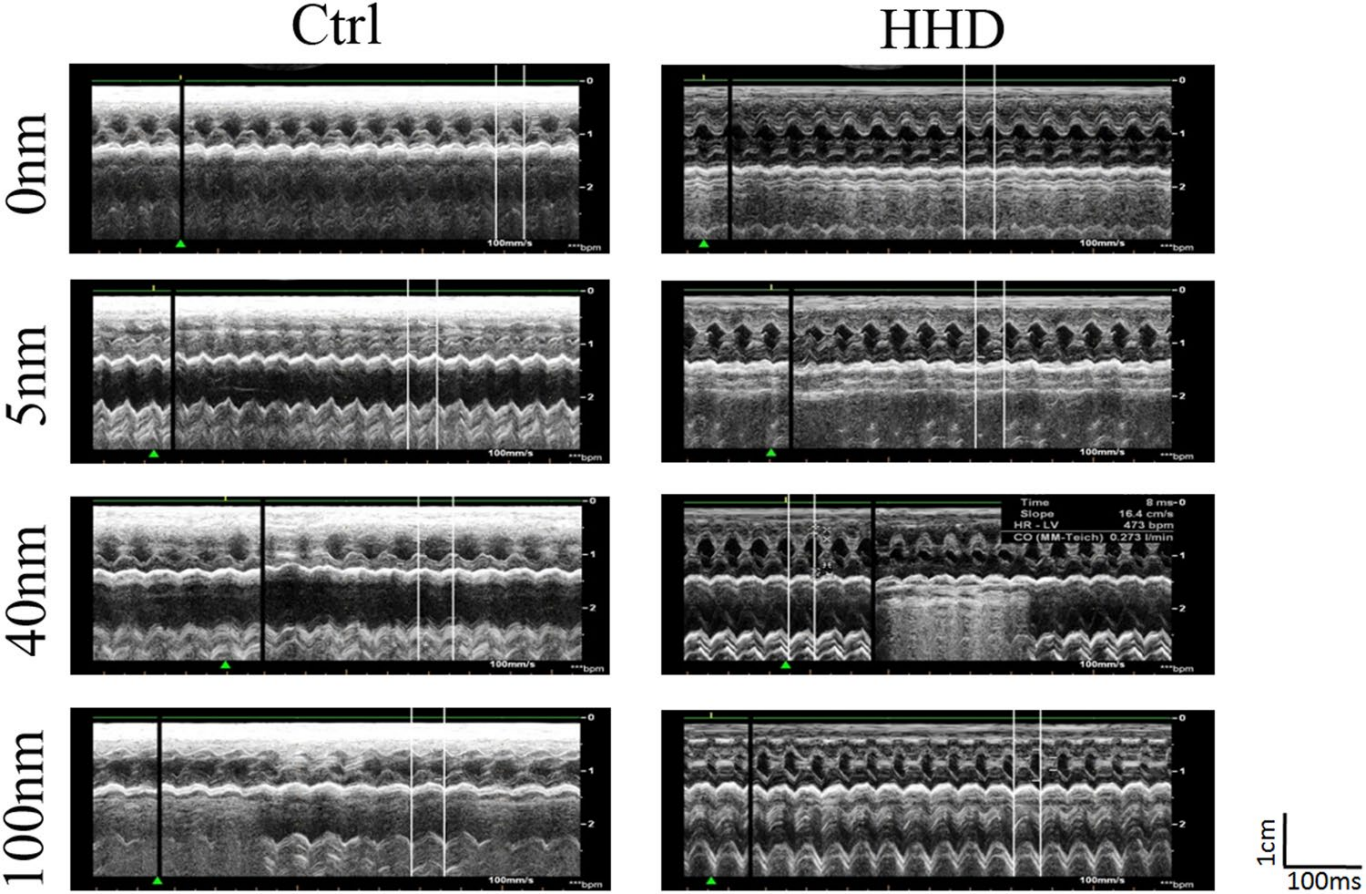
Representative images of echocardiography of rats treated with Au- NPs in normal (A) and ISO-induced hyperthyroid heart disease groups (B). (**A**) Representative images of echocardiography of rats treated with Au-NPs in normal groups; (**B**) Representative images of echocardiography of rats treated with Au-NPs in ISO-induced hyperthyroid heart disease groups.

The effects of Au-NPs on ventricular repolarization function were compared by T wave amplitude and duration and their ratio. Au-NPs had no effect on T wave parameters in control groups (Table 1). Since T wave duration increased in HHD control compared to the normal control (Table 1), and T wave amplitude tended to decrease in HHD groups, T wave morphology in HHD groups was flatter than those in the control groups (Fig. 2, Table 1). This phenomenon was the classic changes of hyperthyroid heart disease, which further confirmed that animal modeling creation was successful. 5 nm-AuNPs decreased T wave in HHD groups which was increased by ISO induction in HHD control groups. These results indicated that 5 nm Au-NPs may protect ventricular repolarization duration from increasing in hyperthyroid heart disease groups.

From the evaluation of the above-mentioned electrocardiogram parameters, it was suggested that 5 nm could lead to protective effect in HHD groups, 40 nm and 100 nm have cardiac toxic potential in HHD groups.

### Au-NPs can affect cardiac function and heart morphology in both normal and hyperthyroid rats

We used echocardiography to evaluate the effects of Au-NPs on both control and ISO-induced hyperthyroid heart disease rats. The representative images of echocardiography treated with Au-NPs in both groups were shown in Fig. 2. All relevant parameters were measured and listed in Table 2.

From echocardiography measurement (Table 2), we found that fractional shortening (FS%), cardiac output (CO), left atrial longitudinal diameter and heart rate (BP) were all increased in ISO-induced hyperthyroid heart disease groups compared with the control groups, which indicated the typical hyperdynamic circulatory state of hyperthyroid heart disease in these rats. The increase in left atrial longitudinal diameter also indicated that ISO injection produced the trend for atrial enlargement (Ctrl-0 nm: 4.52 ± 0.15 vs HHD-0 nm: 4.99 ± 0.28 mm, P > 0.05, n = 6), which is common in long-term hyperthyroidism. All these results indicated large dose of ISO injection for a week successfully created the hyperthyroid heart disease animal model.

The effects of Au-NPs on cardiac function were measured by ejection fraction (EF%), fractional shortening (FS%) and cardiac output (CO, L/min). These parameters are main indicators for heart functions with the EF% as the key one. The decreased value in EF% indicates decreased heart function. Our results showed that 40 nm-AuNPs decreased EF%, and FS% in both control and hyperthyroid groups, but 5 nm and 100 nm Au-NPs had no significant effects on them (Table 2). All sizes of Au-NPs decreased CO in control groups (Table 2), but only 5 nm Au-NPs decreased CO in HHD groups. These results indicated that 40 nm Au-NPs reduced cardiac function in HHD groups.

The effects of Au-NPs on atrial size were evaluated by left atrial cross-sectional and longitudinal diameters. Au-NPs were unable to decrease atrial enlargement in HHD groups. 5 nm-AuNPs decreased left atrial cross-sectional diameter in control groups, indicating the decreased atrial size; while 40 nm Au-NPs increased left atrial cross-sectional diameter in control groups, indicating atrial enlargement (Table 2). These results indicated that 5 nm may has cardiac protective effect while 40 nm may be cardiac toxic.

The effects of Au-NPs on left ventricular dimension and volume were tested by measuring left ventricular end-diastolic diameter (LVEDd), left ventricular end-systolic diameter (LVEDs), End-diastolic volume (EDV) and End-systolic volume (ESV). Au-NPs intervention had no effect on left ventricular dimension and volume in normal groups for 7 days period. 40 nm-AuNPs had the potential to increase LVEDs and to enlarge ESV in HHD groups (Table 2), which was in accordance with the results of electrocardiogram. These results indicated that 40 nm may cause cardiac muscle injury in HHD groups.

The effects of Au-NPs on ventricular thickness were measured by Interventricular Septum Diameter at the end of systole (IVSs) and Interventricular Septum Diameter at the end of diastole (IVSd), Left Ventricular Posterior Wall diameter at the end of diastole (LVPWd) and Left Ventricular Posterior Wall diameter at the end of systole (LVPWs). 100 nm-AuNPs increased LVPWs in control groups (Table 2), indicating 100-AuNPs can exert cardiac hypertrophy in normal hearts.

From the above-mentioned results, we concluded that 5 nm-AuNPs had cardiac protective potential by reducing atrial dimension. 40 nm-AuNPs had apparent cardiac toxic by decreasing cardiac function in both groups. 100 nm-AuNPs had cardiac toxic effect by causing cardiac hypertrophy in control groups. These results were basically in accordance with all measured parameters from electrocardiogram.

### Different Au-NPs exert dynamic changes on lipid profile and cardiac marker enzyme

We tested the effects of Au-NPs on blood lipids in both groups by measuring aspartate transaminase (AST), (total cholesterol) CHOI and (triglyceride) TG. 100 nm-AuNPs increased AST in HHD groups (Table 3), indicating 100 nm may cause liver function damage in HHD groups. 5 nm and 40 nm Au-NPs decreased TG in HHD groups (Table 3), showing that 5 and 40 nm had protective potential toward coronary heart diseases.

**Table 3:** Effects of Au-NPs on cardiac marker enzyme measured in normal and ISO-induced hyperthyroid heart disease groups.

	Group	0 nm	5 nm	40 nm	100 nm
AST(U/L)	Ctrl	261.00 ± 63.61	354.8 ± 77.08	450.33 ± 153.05	301.33 ± 49.70
	HHD	154.75 ± 29.56	168.8 ± 27.92	157.4 ± 12.93*	288.33 ± 56.02*
CHO1(mmol/L)	Ctrl	1.09 ± 0.04	1.14 ± 0.10	1.09 ± 0.10	1.03 ± 0.09
	HHD	1.27 ± 0.21	1.10 ± 0.02	0.96 ± 0.09	0.97 ± 0.06
TG(mmol/L)	Ctrl	0.83 ± 0.20	0.60 ± 0.09	0.7 ± 0.20	0.39 ± 0.03
	HHD	1.08 ± 0.14	0.67 ± 0.07*	0.54 ± 0.14*	0.81 ± 0.06
HDL-C(mmol/L)	Ctrl	0.34 ± 0.03	0.36 ± 0.03	0.4 ± 0.04	0.33 ± 0.005
	HHD	0.31 ± 0.005	0.4 ± 0.03	0.32 ± 0.04	0.33 ± 0.03
LDL-C(mmol/L)	Ctrl	0.34 ± 0.04	0.29 ± 0.04	0.27 ± 0.04	0.3 ± 0.04
	HHD	0.45 ± 0.07	0.3 ± 0.02	0.26 ± 0.02*	0.33 ± 0.05
APOA(g/L)	Ctrl	0.00067 ± 0.001	0.00025 ± 0.00027	0.00125 ± 0.001	0.0002 ± 0.00024
	HHD	0.0005 ± 0.0015	0.00026 ± 0.00037	0.0005 ± 0.001	0.00028 ± 0.00028
APOB(g/L)	Ctrl	0.0067 ± 0.00033	0.0142 ± 0.00217*	0.017 ± 0.001**	0.007 ± 0.001
	HHD	0.007 ± 0.002	0.007 ± 0.00071	0.0065 ± 0.00065	0.006 ± 0.001
LDH(U/L)	Ctrl	875.33 ± 73.44	770.25 ± 94.82	412.0 ± 50.0*	392.0 ± 93.74*
	HHD	1504.0 ± 267.0	1591.5 ± 365.0	904.0 ± 40.1	1449.5 ± 9.5
HBDH(U/L)	Ctrl	337.33 ± 18.32	270.5 ± 27.67	185.0 ± 23.0*	374.0 ± 45.0**
	HHD	501.0 ± 122.0	540.0 ± 123.37	316.25 ± 10.36	474.0 ± 39.0
CK(U/L)	Ctrl	1061.25 ± 16.38	2027.0 ± 62.91*	911.0 ± 41.85	1022.0 ± 16.0
	HHD	748.25 ± 111.57	1751.0 ± 316.15*	780.8 ± 180.83	1045.33 ± 145.83
CKMB(U/L)	Ctrl	683.0 ± 60.34	462.75 ± 41.84*	324.5 ± 24.5*	1108.5 ± 88.5
	HHD	955.0 ± 70.0^§^	1191.75 ± 206.77	619.75 ± 36.77**	952.5 ± 3.5

^*^P < 0.05, **P < 0.01, ***P < 0.001; n = 6.

We further evaluated the effect of Au-NPs on lipid protein and apolipoprotein in both groups. HDL-C (high density lipoprotein-cholesterol), LDL-C (low density lipoprotein-cholesterol), APOA (apolipoprotein A) and APOB (apolipoprotein B) were measured. 40 nm-AuNPs decreased LDL-C in HHD groups (Table 3), indicating 40 nm had cardiac protective effect against coronary heart disease. 5 nm and 40 nm Au-NPs increased APOB level in control groups (Table 3), indicating that 5 and 40 nm Au-NPs had cardiac protective potential toward coronary heart disease. This was in agreeable with one clinical study^16^.

We also tested the effect of Au-NPs on general cardiac marker enzyme, such as Lactate dehydrogenase (LDH) and hydroxybutyrate dehydrogenase (HBDH). The increased HBDH normally occurs in acute heart attack, and the ratio of LDH/HBDH is valuable for diagnose the liver and heart diseases. 40 nm-AuNPs decreased LDH (Table 3) and HBDH in control groups, indicating 40 nm had cardiac protective potential against cardiac muscle injury.

CK-MB (Creatine Kinase Isoenzyme) is the isoenzyme of CK (Creatine kinase) specifically expressed in hearts and skeletal muscle, and they are usually used in clinic to diagnose acute heart infarction. CK-MB will normally increase during the first several days after heart injury, and then CK-MB will decrease after the window period^17^. Therefore, we evaluated the effects of Au-NPs on the heart by this specific cardiac marker enzyme. CK-MB level increased in HHD groups compared with the control groups (Table 3), confirming again the successful of the ISO-induced hyperthyroid heart disease animal model. 5 nm-AuNPs increased CK in both groups but decreased CK’s specific cardiac isoenzyme CK-MB in control groups. 40 nm-AuNPs decreased CK-MB in control and HHD groups (Table 3). Combining these results, it was clearly shown that 5 nm Au-NPs had no cardiac toxic effect. Since 40 nm-AuNPs has been shown to be cardiac toxic by electrocardiogram and echocardiography detections, the abnormally decreased CK-MB by 40 nm did not indicate that 40 nm had cardiac protection effect, rather than that it may cause cardiac toxicity. It might be possible that CK-MB may have increased immediately after 40 nm Au-NPs injection but decreased during following 7 days. The increased CK value also needs to be checked to exclude the possibility of skeletal muscle injury.

We concluded that 5 and 40 nm Au-NPs had cardiac protective effect against coronary heart diseases, since they can decrease harmful lipid or lipid proteins. 5 nm had cardiac protective effect towards cardiac injury (CK-MB decreased in normal groups), and 40 nm Au-NPs may have cardiac toxicity towards cardiac injury which were in accordance with electrocardiogram and echocardiography results. 100 nm Au-NPs processed toxic effect against liver function (decreased AST in HHD groups), while 100 nm Au-NPs didn’t have any effect on CK or CK-MB, indicating that100 nm Au-NPs showed no cardiac protective effects.

### Size-dependent Au-NPs accumulation in the heart is also dependent on the state of disease

**Figure 3:**
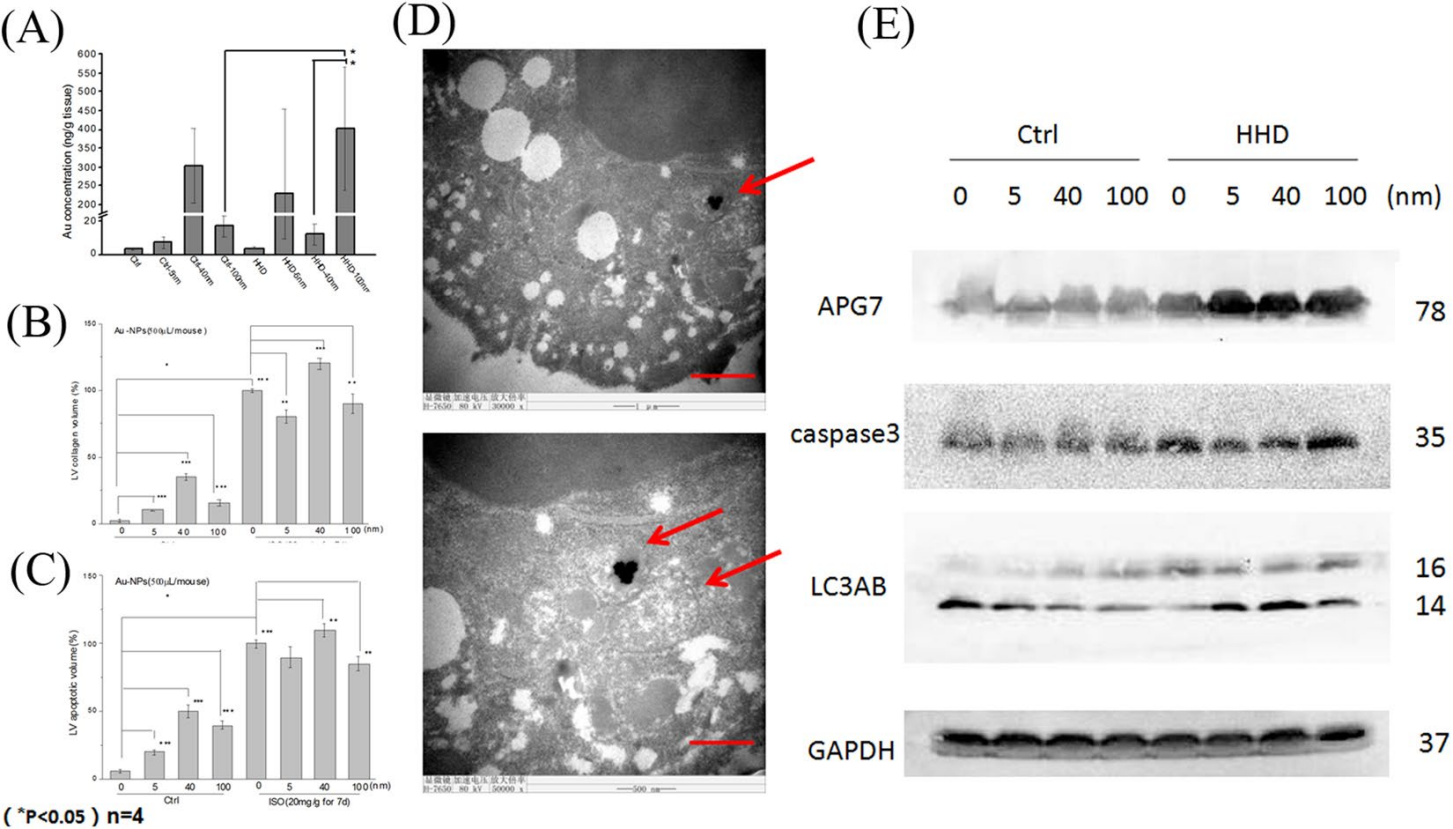
Accumulation of Au-NPs in the heart in control and ISO-induced hyperthyroid heart disease groups. (**A**) Accumulation of Au in the heart in both control and hyperthyroid heart disease groups (scale bar = 1 μm). 100 nm Au-NPs accumulated more in the hearts in HHD groups than in normal groups (Ctrl-100 nm: 16.79 ± 6.16 vs HHD-100 nm: 401 ± 163.41 ng/g heart tissue, P < 0.05, n = 4). 40 nm-AuNPs accumulated less than 100 nm-Au-NPs in the hearts in HHD groups (HHD-40 nm:12 ± 6.37 vs HHD-100 nm: 401 ± 163.41 ng/g tissue, P < 0.05, n = 4). (**B**) MASSON (magnification, x20) staining. More cardiac fibrosis was quantified in the hearts of HHD groups than those in control groups, (Ctrl-0 nm: 2 ± 1.2% vs HHD-0 nm: 100 ± 1.0%, P < 0.001, n = 6). Within control groups, 5 nm Au-NPs (Ctrl-0 nm: 2 ± 1.2% vs Ctrl-5 nm: 10 ± 1.0%, P < 0.001, n = 6), 40 nm Au-NPs (Ctrl-0 nm: 2 ± 1.2% vs Ctrl-40 nm: 35 ± 3.0%, P < 0.001, n = 6) and 100 nm-Au-NPs (Ctrl-0 nm: 2 ± 1.2% vs Ctrl-100 nm: 15 ± 2.3%, P < 0.001, n = 6) caused more fibrosis than normal control which was treated with 0 nm Au-NPs. Within HHD groups however, 5 nm Au-NPs (HHD-0 nm: 100 ± 1.0% vs HHD-5 nm: 80 ± 5.0%, P < 0.01, n = 6) and 100 nm Au-NPs (HHD-0 nm: 100 ± 1.0% vs HHD-100 nm: 90 ± 7.0%, P < 0.001, n = 6) caused less fibrosis than the HHD control which was treated with 0 nm Au-NPs. While  40 nm Au-NPs caused more (HHD-0 nm: 100 ± 1.0% vs HHD-40 nm: 120 ± 4.0%, P < 0.01, n = 6). *P < 0.05; **P < 0.01, ***P < 0.001. (**C**) TUNEL (magnification, x40) staining. More cardiac apoptosis was quantified in the hearts of HHD groups than those in control groups (Ctrl-0 nm: 6 ± 1.1% vs HHD-0 nm: 100 ± 3.1%, P < 0.001, n = 6). 5 nm AuNPs (Ctrl-0 nm: 6 ± 1.1% vs Ctrl-5 nm: 20 ± 2.0%, P < 0.001, n = 6), 40 nm AuNPs (Ctrl-0 nm: 6 ± 1.1% vs Ctrl-40 nm: 50 ± 5.0%, P < 0.001, n = 6) and 100 nm-AuNPs (Ctrl-0 nm: 6 ± 1.1% vs Ctrl-100 nm: 40 ± 3.4%, P < 0.001, n = 6) caused more apoptosis than Ctrl treated with 0 nm Au-NPs. In HHD group however, 100 nm AuNPs (HHD-0 nm: 100 ± 3.1% vs HHD-100 nm: 85 ± 5.3%, P < 0.01, n = 6) caused less apoptotic volume than HHD treated with 0 nm Au-NPs. While 40 nm Au-NPs caused more (HHD-0 nm: 100 ± 3.1% vs HHD-40 nm: 110 ± 4.6%, P < 0.001, n = 6). (n = 4 to ≈6). *P < 0.05; **P < 0.01, ***P < 0.001. (**D**) Transmission electron microscopy observation H9c2 cells were seeded in T25 (1 × 10^6^ cells/mL) and cultured for 24 hr with different sizes of Au-NPs at a concentration of 10 µg/mL. Representative upper image (scale bar = 1 μm) showed that 100 nm Au-NPs undergone typical autophagy process in the cells. Lower image showed that (scale bar = 0.6 μm) Au-NPs were enwrapped in a phagocytic vesicle and they were going to fuse with lysosome. (**E**) Representative images of Western blotting showing the expression levels of APG7, caspase 3, LC3A/B and GAPDH. Quantification of APG7, caspase 3, LC3A/B levels indicated that 40 and 100 nm Au-NPs cause autophagy in control group while 5,40 and 100 nm Au-NPs elevated autophagy in HHD groups (n = 6). *P < 0.05.

To explore the reasons for the different effects of Au-NPs on control and HHD rats, we tested the accumulation of Au-NPs in both control and hyperthyroid heart disease rats. Au-NPs accumulation turned to be animal state dependent. 100 nm Au-NPs accumulated more in the hearts of the HHD groups than those in control rats (Ctrl-100 nm: 16.79 ± 6.16 vs HHD-100 nm: 401 ± 163.41 ng/g tissue, P < 0.05, n = 4, Fig. 3A). Different sizes of Au-NPs also accumulated differently in the HHD hearts. 40 nm-AuNPs accumulated less than 100 nm-AuNPs in the hearts of the HHD groups (HHD-40 nm:12 ± 6.37 vs HHD-100 nm: 401 ± 163.41 ng/g heart tissue, P < 0.05, n = 4, Fig. 3A).

### Au-NPs markedly exaggerate cardiac fibrosis and apoptosis in normal rats but decrease histological alteration in ISO-induced hyperthyroid heart disease groups in size-dependent manner

**Figure 4:**
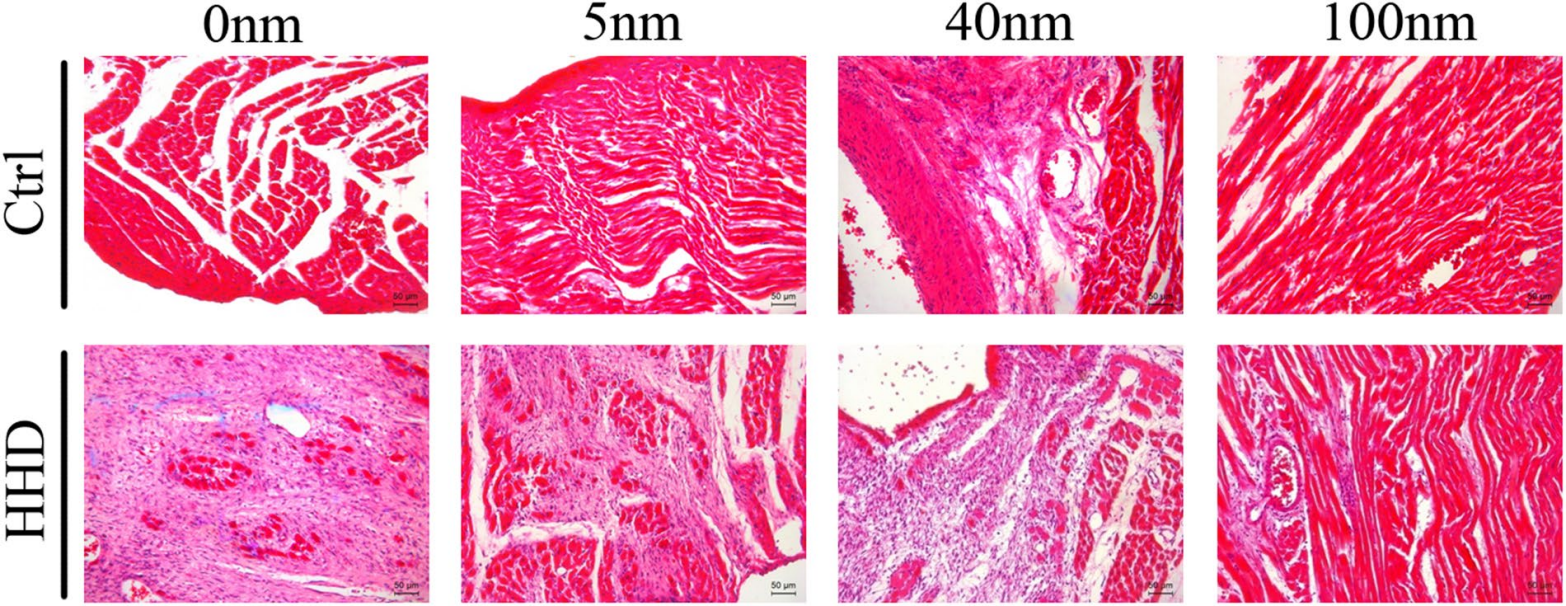
The effects of Au-NPs on cardiac histological changes pronounced by HE stain in normal and ISO-induced hyperthyroid heart disease groups. HE (magnification, x20) staining was performed to determine general histological alterations in control and ISO-induced hyperthyroid heart disease groups. Red part represented muscle cytoplasm while blue part demonstrated nucleus in HE stains. The histological heart alterations induced by Au-NPs were size-dependent with 40 nm AuNPs demonstrated prominent congested dilated blood vessels in control groups, while for HHD groups, the pathological changes were decreased with few lymphocytic infiltrate and less disturbed muscle fascicles when combined with 5 nm and 100 nm application (n = 6).

We further tested if there are histological alterations by Au-NPS in both normal and HHD rats. HE, hematoxylin-eosin staining (magnification, x20) staining was performed to determine general histological alterations in control and ISO-induced hyperthyroid heart disease groups (Fig. 4). Red part represented muscle cytoplasm while blue part demonstrated nucleus in HE stains. The histological heart alterations induced by Au-NPs were size-dependent with 40 nm Au-NPs demonstrated prominent congested dilated blood vessels in control groups, while for HHD groups, the pathological changes were decreased with few lymphocytic infiltrate and less disturbed muscle fascicles when combined with 5 nm and 100 nm Au-NPs application (n = 4 to 6).

**Figure 5:**
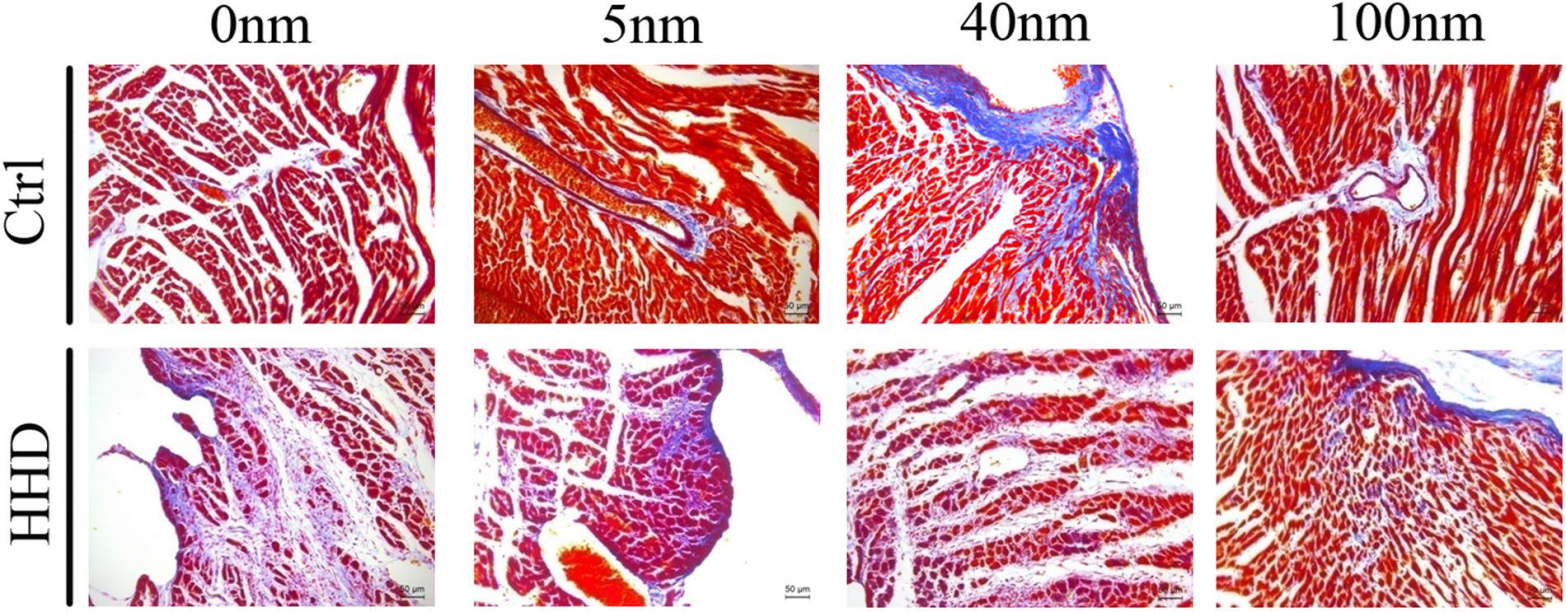
The effects of Au-NPs on cardiac left ventricular collagen volume pronounced by Masson stain in normal and ISO-induced hyperthyroid heart disease groups. Masson (magnification, x20) staining was performed to determine cardiac fibrosis in control and ISO-induced hyperthyroid heart disease groups. Red part represented muscle tissue while blue part demonstrated fibrosis in Masson stain. The fibrosis volume induced by Au-NPs was size-dependent with 40 nm AuNPs induced more effects in control groups, while for ISO-induced hyperthyroid heart disease groups, more pronounced fibrosis volume was caused but decreased when combined with 5 nm and 100 nm AuNPs application (n = 6).

MASSON (magnification, x20) staining was performed to determine cardiac fibrosis of the hearts in two groups treated with different sizes of Au-NPs (Fig. 5). Quantification results showed that there was more cardiac fibrosis in the hearts of HHD groups than those in control groups, (Ctrl-0 nm: 2 ± 1.2% vs HHD-0 nm: 100 ± 1.0%, P < 0.001, n = 6, Fig. 3B). Within control groups, 5 nm Au-NPs (Ctrl-0 nm: 2 ± 1.2% vs Ctrl-5 nm: 10 ± 1.0%, P < 0.001, n = 6), 40 nm Au-NPs (Ctrl-0 nm: 2 ± 1.2% vs Ctrl-40 nm: 35 ± 3.0%, P < 0.001, n = 6) and 100 nm-Au-NPs (Ctrl-0 nm: 2 ± 1.2% vs Ctrl-100 nm: 15 ± 2.3%, P < 0.001, n = 6) caused more fibrosis than normal control which was treated with 0 nm Au-NPs (Fig. 3B). Within HHD groups however, 5 nm Au-NPs (HHD-0 nm: 100 ± 1.0% vs HHD-5 nm: 80 ± 5.0%, P < 0.01, n = 6) and 100 nm Au-NPs (HHD-0 nm: 100 ± 1.0% vs HHD-100 nm: 90 ± 7.0%, P < 0.001, n = 6) caused less fibrosis than the HHD control which was treated with 0 nm Au-NPs, while 40 nm Au-NPs caused more (HHD-0 nm: 100 ± 1.0% vs HHD-40 nm: 120 ± 4.0%, P < 0.01, n = 6). (Fig. 3B). *P < 0.05; **P < 0.01, ***P < 0.001.

**Figure 6:**
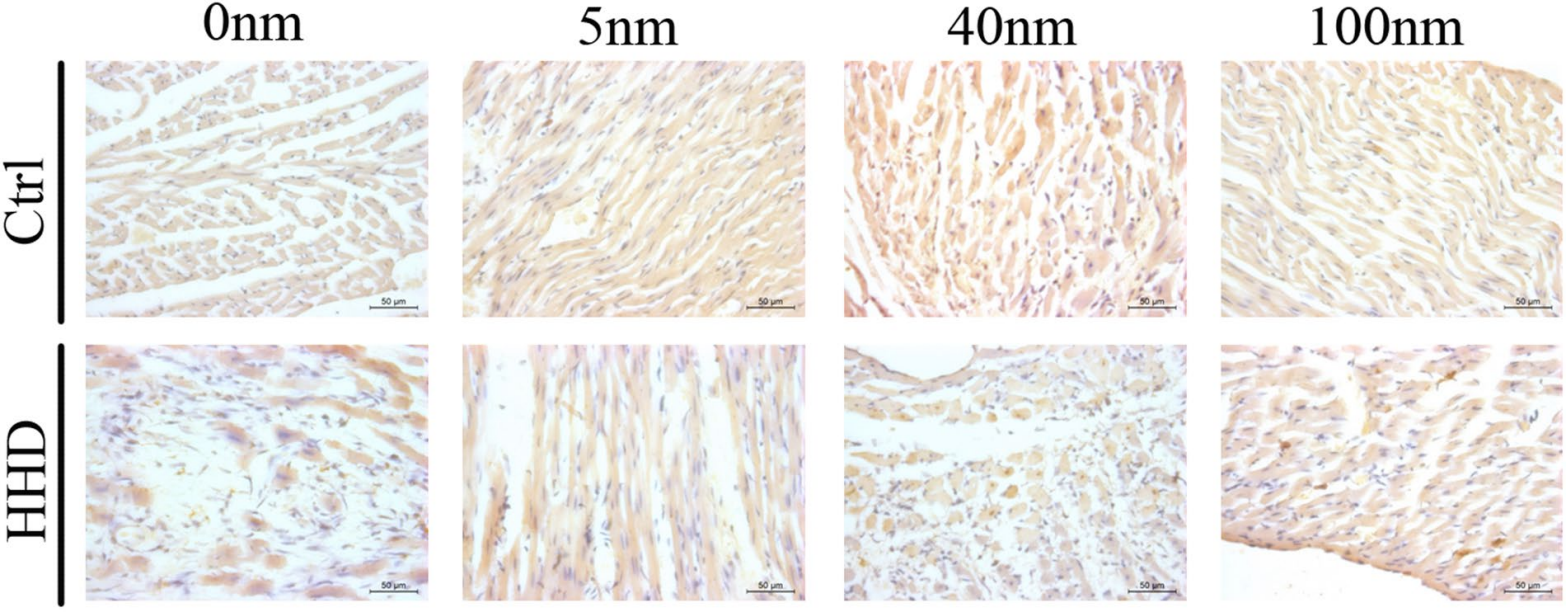
The effects of Au-NPs on cardiac left ventricular apoptotic volume by TUNEL analysis in normal and ISO-induced hyperthyroid heart disease groups. Representative images of the TUNEL analysis from the control and ISO-induced hyperthyroid heart disease groups were demonstrated (magnification, x40). Brown nucleus represented apoptotic cells while blue nucleus demonstrated normal cells in TUNEL stain. The apoptotic volume induced by Au-NPs was size-dependent with 40 nm AuNPs  induced more effects in control groups, while for ISO-induced hyperthyroid heart disease groups, more pronounced apoptotic volume was caused but decreased when combined with 100 nm AuNPs application (n = 6).

TUNEL (magnification, x40) staining was performed to determine cardiac apoptosis of the hearts in two groups treated with different sizes of Au-NPs (Fig. 6). Quantification results show that more cardiac apoptosis was observed in the hearts of HHD groups than those in control groups (Ctrl-0 nm: 6 ± 1.1% vs HHD-0 nm: 100 ± 3.1%, P < 0.001, n = 6) (Fig. 3C). Within control groups, 5 nm Au-NPs (Ctrl-0 nm: 6 ± 1.1% vs Ctrl-5 nm: 20 ± 2.0%, P < 0.001, n = 6), 40 nm Au-NPs (Ctrl-0 nm: 6 ± 1.1% vs Ctrl-40 nm: 50 ± 5.0%, P < 0.001, n = 6) and 100 nm-Au-NPs (Ctrl-0 nm: 6 ± 1.1% vs Ctrl-100 nm: 40 ± 3.4%, P < 0.001, n = 6) caused more apoptosis than those in control group which was treated with 0 nm Au-NPs (Fig. 3C). Within HHD groups however, 100 nm Au-NPs (HHD-0 nm: 100 ± 3.1% vs HHD-100 nm: 85 ± 5.3%, P < 0.01, n = 6) caused less apoptotic volume than those in HHD control which was treated with 0 nm Au-NPs, while 40 nm Au-NPs caused more (HHD-0 nm: 100 ± 3.1% vs HHD-40 nm: 110 ± 4.6%, P < 0.001, n = 6). (Fig. 3C). *P < 0.05; **P < 0.01, ***P < 0.001.

### Autophagy in rat cardiomyocytes treated with Au-NPs and its possible mechanism

To explore the possible mechanism of the effects of Au-NPs, we carried out transmission electron microscopy observation on rat cardiomyocytes H9c2 cells *in vitro*. H9c2 cells were seeded in T25 (1 × 10^6^ cells/mL) and cultured for 24 hr with different sizes of Au-NPs at a concentration of 10 µg/mL. Representative upper image (Fig. 3D, scale bar = 1 μm) showed that 100 nm Au-NPs undergone typical autophagy process in the cells. Lower image showed that (Fig. 3D, scale bar = 0.6 μm) Au-NPs were enwrapped in a phagocytic vesicle and they were going to fuse with lysosome.

Western blotting results (Fig. 3E) showed that the expression levels of APG7, caspase 3, LC3A/B and GAPDH. Quantification of APG7, caspase 3, LC3A/B levels indicated that 40 and 100 nm Au-NPs caused autophagy in control groups while 5, 40 and 100 nm Au-NPs elevated autophagy in HHD groups (n = 6). *P < 0.05.

## Discussion

End-stage of hyperthyroid heart disease and many other heart diseases is heart failure, which has high morbidity and mortality and poor diagnosis. Currently most patients with heart diseases need to take various drugs for their life time. Since the diverse side-effects of those drugs, the patients’ compliance to treatment are low, and this leads to high re-hospitalization. NPs have gained positive results in phase III cancer treatment due to its good targeting and delivery ability, but there are only limited studies of using Au-NPs in the treatment of heart diseases *in vitro*. It is also known that the characteristics of nanoparticles can change dramatically between *in vivo* and *in vitro*. Therefore *in vivo* studies are fundamentally needed before Au-NPs application can be considered in clinical use.

In this study, firstly we created an ISO-induced hyperthyroid heart disease animal model. After 7 days of single dose intraperitoneal injection in normal rats and we created ISO-induced hyperthyroid rats, we then tested cardiac effects of different sizes of Au-NPs by measuring various parameters from electrocardiogram, echocardiography and cardiac marker enzymes *in vivo/in vitro*.

Hyperthyroid heart disease is generated by the effect of excess thyroid hormones on thyroid hormone receptors which is important for control of heart rate^18^. The high sensitivity of this heart rate controlling receptor of thyroid hormones or excessive high level of thyroid hormones can cause excessive fast heart rate. That is the mechanism or pathophysiological of hyperthyroid heart disease. If heart rate remains fast for a longer time, for example several days or weeks, the subject will present with the typical manifestation of hyperthyroid heart disease, such as high cardiac output and typical atrial ECG changes as described in our Echocardiography and ECG results (Figs 1, 2). Our hyperthyroid heart disease model was produced by consecutive isoproterenol injection for a week. It is well known that isoproterenol can target specifically of the ß1-receptor which mainly expressed in the heart, and the activation of this receptor can cause heart rate increase. Although there are other studies using levothyroxine to induce hyperthyroid heart disease model, but it usually takes 6 to 7 weeks, much longer than our 1-week period^19^, and those studies mainly focused on the endocrine secretion rather than on hyperthyroid heart disease. Therefore, we used ISO to induce the hyperthyroid model rather than the traditional levothyroxine method, and all measured parameters proved that our model was successful and was good to be used to test the effects of Au-NPs in hyperthyroid heart disease.

Our results showed that Au-NPs of 40 nm showed cardiac toxicity in both control and HHD groups, that 100 nm Au-NPs showed cardiac toxicity mainly in control groups, and that 5 nm Au-NPs showed no cardiac toxicity. 100 nm Au-NPs accumulated more in hyperthyroid rat hearts than in the normal rat hearts. These results indicated that the cardiac protective effect of NPs does not entirely rely on the accumulation of Au in hearts.

We recorded electrocardiogram and echocardiography 7 days after single dose of 10 ug of Au-NPs administration. Our results showed that 40 nm-AuNPs reduced cardiac function in both normal and HHD groups. In addition, 40 nm-AuNPs caused atrial enlargement in HHD groups. On the other hand, 100 nm-AuNPs increased LVPWs in control groups, indicating 100-AuNPs can cause cardiac hypertrophy in normal hearts, while 5 nm-AuNPs had cardiac protective potential by reducing atrial dimension.

There is no report about cardiac toxicity by single dose of Au-NPs administration. In our study, single dose administration of 40 nm and 100 nm Au-NPs can induce cardiac toxicity after 7 days while 5 nm has no cardiac toxicity. Therefore, we concluded that the cardiac toxicity is dependent on the size of Au-NPs, and smaller size of Au-NPs may have little cardiac toxicity *in vivo*. Yang *et al*. used 13 nm Au-NPs to treat ISO (5 mg/kg)-induced cardiac hypertrophy rats and showed that Au-NPs induced no cardiac protective and toxicity effect^20^. Another study^21^ showed that the repeated administration of 10 nm-AuNPs *via* tail veins for 14 consecutive days can induce cardiac hypertrophy in normal mice, but this effect can be reversed during washout period. Taken together that 5 nm has no cardiac toxicity in our study, we inferred that Au-NPs ranging from 10 nm to 20 nm would be possible candidate to be used in treating heart disease. We suspect that small size Au-NPs might have cardiac toxicity if administrated by repeated dose with much longer exposure time^22,23^. Further studies are needed to confirm this. Other small sizes of Au-NPs, either <5 nm or any between 5 to 40 nm also need to be chosen to carry out same test.

Moreover, in biochemical test of blood taken from rats of both groups, we found that CK-MB was decreased in both 40 nm-AuNPs treated control groups and HHD groups. Electrocardiography results has already showed that 40 nm Au-NPs have cardiac toxicity, and the CK-MB were only detectable after several days of cardiac damage. Therefore 40 nm-AuNPs had cardiac toxicity, but this effect may not be detected by cardiac enzyme parameters 7 days after Au-NPs were injected. However, Ferreira *et al*. showed that Au-NPs (10 and 30 nm) can lead to oxidative damage several weeks after the acute administration for a single intraperitoneal injection^24^, therefore more persistent and specific cardiac marker enzyme need to be developed to delicately measure the exact toxicity of Au-NPs on hearts.

Secondly, we studied the relationship between toxicity effects and distribution and accumulation of Au-NPs in normal and diseased hearts. In our study, different sizes of Au-NPs were injected intravenously in rats. After 7 days of ISO intervention in HHD groups, we took hearts from both groups to measure the concentration of Au by inductively coupled plasma-mass spectrometry (ICP-MS). Our results showed that in control groups without ISO injection, 40 nm-AuNPs showed the highest Au accumulation, while 100 nm ranked the second, 5 nm the least. These results are similar to one previous study, in which ICP-MS detection showed that small Au-NPs (13 nm) accumulated higher levels in heart compared with 4 nm and 100 nm after 7 days injection^25,26^.

In HHD groups, 100 nm-AuNPs accumulated most in hearts, while 40 nm ranked the second and 5 nm the least. It is reported that larger NPs can accumulate in diseased heart better than in normal heart. Lundy *et al*. found that 20–200 nm diameter nanoparticles are optimal for passive targeting of the injured left ventricle immediately following cardiac ischemia-reperfusion injury in mice^27^. This is in accordance with our study which showed that 100 nm can target the hearts of HHD groups better than the smaller sizes. However, our data also showed that 100 nm-AuNPs can induce cardiac hypertrophy in hyperthyroid heart disease rats. Therefore, cautions are needed when applying larger size NPs.

Finally, our study showed that 5 nm-AuNPs decreased left atrial cross-sectional diameter in control groups, which means that 5 nm-AuNPs may process cardiac protective potential to decrease atrial enlargement. Previous studies^28^ reported that ultra-small NPs are cardiac toxic, since they can block hERG channels *in vitro*, which means they have high risk of cardiac arrhythmia. However, further *in vivo* analysis showed that high dose of 50 mg/kg 1.4 nm AnNPs did not block the channel and give a trace of heart arrhythmia. NPs can form a protein corona when confronted with protein-containing solutions like serum or full blood^29,30^, in which process will lead to the enlargement of the diameter of NPs. Since there is no *in vivo* study of smaller NPs size than 10 nm in treating heart disease, our results confirmed that although 5 nm-AuNPs didn’t accumulate as much as 40 nm and 100 nm Au-NPs did in hearts, they had cardiac protective potential.

Histological analysis and TUNEL staining showed that Au-NPs can induce pathological alterations including cardiac fibrosis, apoptosis in control group, however they can protect HHD groups from these harmful effects. Further transmission electron microscopy using H9C2 cells (a sub-clone derived from embryonic rat heart tissue) *in vitro* showed that autophagy presented in Au-NPs treated cells. In this case, Western blotting focusing on autophagy process in Au-NPs treated mouse were conducted to evaluate the effect of autophagy. The results showed that Au-NPs can suppress (microtubule-associated protein 1 light chain 3) LC3 I turning to LC3 II which is a standard phenomenon of autophagy^31,32^, and decrease autophagy-associated protein APG7^33^ and apoptosis-associated protein caspase 12^34^ in the process in HHD groups but presented opposite effects on control groups. It is known that autophagy can trigger apoptosis and lead to cell death, but it can also play cardiac beneficial role in the heart^35^. Therefore, we conclude that Au-NPs can induce protective effect on HHD groups by decreasing their autophagy level, but increase autophagy in control groups, which could be an adaptive inflammation reacts. However, they can induce cardiac toxicity^36^ and change the cardiac function if their uses are not properly controlled. These effects of Au-NPs on heart can bring altered histological structure in the first place before they can change the cardiac function.

Au-NPs are known to have antioxidant potential in treating disease^15^. Most of intracellular reactive oxygen species (ROS) are generated as by-products of mitochondrial bioenergetics. Excess ROS production in heart muscle is one of the mechanism and indicator for heart disease progression^37^. Au-NPs might exert its cardiac effects through changing the level of ROS production. We also observed that Au-NPs were involved with autophagy when they are entering rat heart muscle cells *in vitro*. It is known that autophagy can either induce cell survive or cell death. Under certain circumstances, autophagy constitutes a stress adaptation that avoids cell death (and suppresses apoptosis), whereas in other cellular settings, it constitutes an alternative cell-death pathway^34^. Therefore, it is possible that Au-NPs can change the state of cells from autophagy to apoptosis or *vice versa*. Further studies are needed to evaluate these possible mechanisms.

In conclusion, our study showed that effects of Au-NPs on heart is size dependent. This might be linked to their size-dependent accumulation and distribution in hearts. The larger sizes of Au-NPs (more than 40 nm) are more likely to have cardiac toxicity effect. Thus, we recommend using smaller size of Au-NPs (<40 nm) in the treatment of the heart diseases. Moreover, although 5 nm didn’t accumulate in hearts as much as larger sizes in both normal and diseased hearts, it induced cardiac protective potential otherwise. The protective effect of Au-NPs need to be carefully monitored in relevant heart disease state before its effective clinical use.

As there are few animal studies about using nanoparticles in the treatment of heart disease, our *in vivo* and *in vitro* studies would provide valuable information before they can be considered for clinical use in general.

## Materials and Methods

### Gold nanoparticles

Au-NPs of different sizes (5, 40, 100 nm) were purchased from sigma. (Code: 752568, 741981, 797758). All Au-NPs used in this study were in PBS solution at a concentration of 0.1 mM. The mean size and morphology of these Au-NPs were evaluated from transmission electron microscope (TEM) as we did before, and they were on within the range of product specification (data not shown).

### Animals

Fifty-six healthy 6–7 weeks old, weight between 250–270 g male *Sprague-Dawley* (SD) rats were obtained from the Laboratory Animal Center of Xi’an Jiaotong University. The use of animal was approved by Xi’an Jiaotong University Ethics Committee (NO: XJTULAC2017-703).

Animals were randomly divided into 8 groups, 4 groups were 0, 5, 40, 100 nm Au-NPs treated control groups and another 4 groups were 0, 5, 40, 100 nm Au-NPs treated hyperthyroid heart disease (HHD) groups. The hyperthyroid heart disease groups were achieved by intraperitoneal injection with ISO (20 mg/kg/mL) from day 0 daily for 7 days. The 5, 40 and 100 nm Au-NPs were administered intravenously from caudal vein with dose of 0.1 mM for 500 µL per rat (that is 10 µg of Au-NPs per rat) at day 0 for all groups. The normal control group (0 nm Au-NPs groups) were injected with normal saline solution (0.9% concentration) with the same amount as ISO treated groups. The initial injections were carried out together at day 0, then the injection was kept at same time each day, therefore the interval time was 24 hrs.

The rats were maintained with standard laboratory rodent diet pellets and housed in humidity and temperature-controlled ventilated cages on a 12 h day/night cycle. All experiments were conducted in accordance with the guidelines approved by Laboratory Animal Care Committee (License No: 7-703).

### Echocardiography

The rats were sedated with 10% chloral hydrate with dosage of 0.3 mL/per 100 g body weight through intraperitoneal injection before recording. Echocardiographic examinations were performed on an IE33 system (Philips, Eindhoven, Netherlands). Cardiac hemodynamics was evaluated as we previously described^29^. Cardiac function parameters such as left ventricular fractional shortening (LVFS), ejection fraction (EF) and cardiac output (CO) were recorded. The parameters evaluating the heart structure, including interventricular septum thickness at the end of systole (IVSTs), and interventricular septem thickness at the end of diastore (IVSTd) were also recorded.

### Electrocardiogram

Animal sedation is the same as above for echocardiography. Electrocardiogram was assessed as we described previously^35^. Electrocardiogram parameters were recorded using BL-420 biological and functional experimental system (Tai Meng, LinYin, Chengdu). All data were analyzed with BL-New Century software (Tai Meng).

### Serum parameters measurement

5 mL of blood were taken from abdominal aorta on day 7 from each animal, and serum were obtained. Normal standard clinical parameters were evaluated by Hitachi LST008 automatic biochemical analyzer (Japan) at the Clinical Laboratory Department of First Affiliated Hospital of Xi’an Jiaotong University. Lipid profile were measured with aspartate aminotransferase (AST), CHOI (total cholesterol), TG (triglyceride), HDL-C (high density lipoprotein), LDL-C (low density lipoprotein), APOA (apolipoprotein A) and APOB (apolipoprotein B). Cardiac enzyme markers including LDH (lactate dehydrogenase), HBDH (hydroxybutyrate dehydrogenase), CK (creatine phosphokinase), CK-MB (Creatine Kinase Isoenzyme) were also evaluated.

### ICP-MS measurement

7 days after single intravenously injection of 10 ug of different sizes of Au-NPs on day 0, animals are sedated as the same as above. Hearts were excised by thoracotomy as described previously^36^. The process of ICP-MS test was carried out as described previously^25^. Briefly, high concentration of HNO_3_ was used to digest the hearts. Then HNO_3_ solutions containing hearts were evaporated. Finally, 2% of HNO_3_ was used to dilute the remains to a 10 ml stock. ICP-MS was used here to evaluate the accumulation of Au in the stock.

### Histological Analysis

For histological analysis, hearts were arrested with a 10% potassium chloride solution at end diastole and then fixed in 4% paraformaldehyde. Fixed hearts were embedded in paraffin and cut transversely into 5-μm sections. Serial heart sections were stained with hematoxylin-eosin or wheat germ agglutinin (No. W11261; Invitrogen) to measure myocyte cross-sectional areas. The degree of collagen deposition was detected by a Masson kit (No. 017756; Promega). The degree of apoptosis was detected by a TUNEL kit (No. ab206386; Abcam). Images were analyzed using a quantitative digital image analysis system (Image-Pro Plus 6.0).

### Western blot analysis

Antibodies against APG7 (No. 8558), LC3A/B (No. 12741), caspase-3 (No. 9662) and GAPDH (No. 2118) were purchased from Cell Signaling Technology (Boston, MA), and horseradish peroxidase-conjugated anti-rabbit IgG and anti-mouse IgG were purchased from Abcam (Cambridge, MA). Proteins were detected using Western blot analysis as described previously^24^. Cellular proteins were extracted according to the manufacturer’s instructions (Santa Cruz, CA). Equal amount of proteins were separated on a 10% SDS-PAGE gel and transferred onto a PVDF membrane (Millipore,MA), which was then incubated with primary antibodies and then with secondary antibodies. The membrane was developed, and protein signals were detected using chemiluminescence. Image acquisition tools and image processing software packages used was Quantity One (provided by Bio-Rad Technology).

### H9c2 cell culture

The rat cardiomyocytes cell line was kindly provided by Jianjun Mu from Xi’an Jiaotong University and cultured as previously described^37^. Briefly, the cells were maintained in DMEM (Hyclone, GE healthcare, USA) supplemented with 10% FBS (Gibco), 100 U/mL penicillin, and 100 µg/mL streptomycin solution (Hyclone, GE healthcare, USA), and then incubated at 37 °C in a 5% CO_2_ humidified atmosphere.

### Transmission electron microscopy observation

H9c2 cells were seeded in T25 (1 × 10^6^ cells/mL) and cultured for 24 hr with different sizes of Au-NPs at a concentration of 10 µg/mL. H9c2 cells were then digested by 0.25% Trysin (Gibco) and harvested by centrifuging at the speed of 1000 rpm for 3 min. The samples were fixed in 2.5% glutaraldehyde followed by 1% perosmic acid and dehydrated in gradual ethanol series. Ultrathin sections (800 nm, thickness) were cut and double-stained with uranyl acetate and lead citrate. The ultra-structures were observed using a Transmission electron microscopy (TEM, HITACHI-H7650, Tokyo, Japan). The sample layer to quantify the intracellular accumulation of Au-NPs was not stained in order to avoid the interference of dye lead and these images were magnified to 4000 times. The sample layer to locate the intracellular Au-NPs were stained with lead dye in order to find out the interaction of Au-NPs with cytoplasmic organoids of rat cardiomyocytes and these images were magnified to 50,000 times.

### Statistics

All data were expressed as the mean ± the standard error of the mean (S.E.M). A one-way ANOVA or two-tailed Student’s t-test was used to determine statistical significance between the control and test groups. A P-value of 0.05 or less was considered significant.

## Electronic supplementary material

Supplementary Materials
