# Supplementary material for: Size dependent effects of Gold Nanoparticles in ISO-induced Hyperthyroid Rats
Source: Sci Rep. 2018 Jul 19;8:10960. doi: 10.1038/s41598-018-27934-9 (PMC6053462; doi:10.1038/s41598-018-27934-9)

Supplementary Materials

Size dependent effects of Gold Nanoparticles in ISO-induced Hyperthyroid Rats

Jingwen Zhang[1#], Yanbo Xue[1#], Yajuan Ni[2], Feifei Ning[1*], Lijun Shang[3*], Aiqun Ma[1*]

[1] Department of Cardiovascular Medicine, First Affiliated Hospital of Xi'an Jiaotong University; Shaanxi Key Laboratory of Molecular Cardiology; Key Laboratory of Environment and Genes Related to Diseases, Ministry of Education, Xi'an, Shaanxi, China; [2]Department of Cardiovascular Medicine, Second Affiliated Hospital of Xi'an Jiaotong University, Xi'an, Shaanxi, China; [3]School of Medical Sciences, University of Bradford, Bradford, BD7 1DP, UK

[#] JZ and YX contribute equally to this article as First Author;

* Corresponding authors: LS [l.shang1@bradford.ac.uk], AM [maaiqun@medmail.com.cn] and FN [ning.feir@foxmail.com].

Au-NPs can affect body weight in rats simultaneously but has limited effects for long periods of time

We first tested if Au-NPs had any effect on body weight of both normal and HHD rats. This provided a valuable and easy way to evaluate the state of rats treated with Au-NPs and was summarized in Fig S1. Au-NPs decreased body weight in HHD rats simultaneously but has limited effects over long periods of time and had no effects in normal groups (0nm-treated group). The body weight in the normal control group increased steadily for 7 days (Ctrl-0nm: first day: 271.40±3.64 mg vs 7th day 300.70 ±3.80 mg, $p<0.001$, n=10) while the body weight in Au-NPs treated control group had no changes (HDD-0nm: Day 1: 280.00±12.33 vs Day 7: 283.50±13.05 mg, $p>0.05$, n=6). However, body weight in hyperthyroid heart disease (HHD) groups decreased at the first day. For example, after Au-NPs injection, the body weight at day 2 was significantly decreased compared to that at day1; for 5nm-AuNPs (HHD-5nm: Day 1: 273.57±4.61 *vs* Day 2: 264.57±5.47 mg, $p<0.05$, n=7); 40nm-AuNPs (HHD-40nm: Day 1: 267.14 ± 5.75 *vs* Day 2: 266.71 ± 7.40 mg, $p>0.05$, n=7); 100nm-AuNPs (HHD-100nm: Day 1: 263.00±5.47 *vs* Day 2: 264.57± 5.29 mg, $p>0.05$, n=7). However, from Day 3 to Day 7, the body weight can increase in all HHD groups except 100nm treated (HHD-0nm: Day 2: 261.00±7.94 *vs* Day 7: 283.20±8.48 mg, $p<0.05$, n=5), (HHD-5nm: Day 2: 262.00±5.71 *vs* Day 7: 287.83 ±7.00 mg, $p<0.05$, n=6) and (HHD-40nm: Day 2: 266.71±7.40 *vs* Day 7: 278.86± 8.53 mg, $p<0.05$, n=7). Although the body weight changed in HHD groups after

Au-NPs injection, it increased eventually, meaning that Au-NPs has biocompatibility. However, the body weight did not change much in the normal groups and the body weights were falling within the range of acceptable variations. There was no observation of significant changes on animal behaviors and body weights (Supplementary Fig 1). This suggested that applying Au-NPs in healthy subjects will not affect their body weight and has limited effect on diseased rats as well.

We must admit that perhaps we need to do bit of selection to better control the body weight measurement in the future experiments. But this information won't be essential in the discussion of their electrophysiological properties.

Figure S1 Effects of Au-NPs on body weight

A: Effects of Au-NPs on the body weight in normal rats. **B:** Nanoparticles effects on the body weights of ISO-induced hyperthyroid rats. Au-NPs decreased body weight in HHD groups simultaneously at day 2 but had limited effects over long periods of time and had no effects in normal groups (0nm-treated groups).

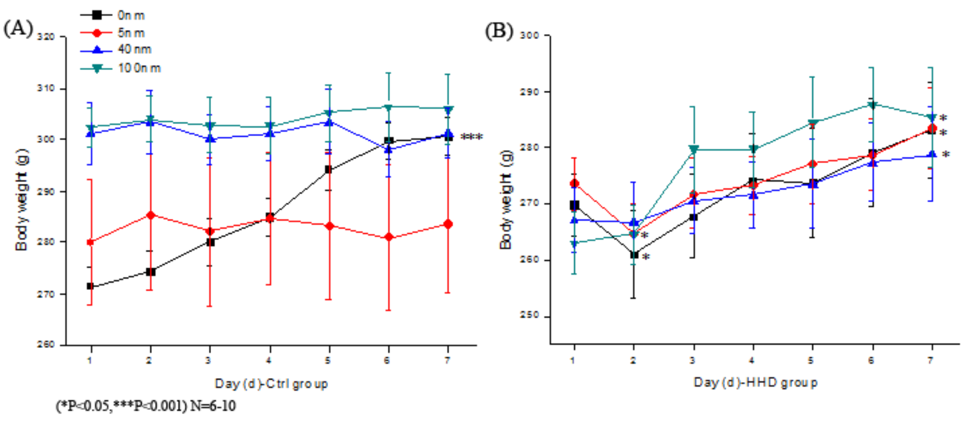

Supplement: Supplementary file 1 — Supplementary Materials [file 41598_2018_27934_MOESM1_ESM.pdf]
